# Anti-complement 5 antibody ameliorates antibody-mediated rejection after liver transplantation in rats

**DOI:** 10.3389/fimmu.2023.1186653

**Published:** 2023-06-16

**Authors:** Tetsuya Tajima, Koichiro Hata, Jiro Kusakabe, Hidetaka Miyauchi, Joshua Sam Badshah, Shoichi Kageyama, Xiangdong Zhao, Sung-Kwon Kim, Tatsuaki Tsuruyama, Varvara A. Kirchner, Takeshi Watanabe, Shinji Uemoto, Etsuro Hatano

**Affiliations:** ^1^ Division of Hepato-Biliary-Pancreatic Surgery and Transplantation, Department of Surgery, Graduate School of Medicine, Kyoto University, Kyoto, Japan; ^2^ Department of Surgery , Division of Abdominal Transplantation, Stanford University School of Medicine, Stanford, CA, United States; ^3^ Alexion Pharmaceuticals Inc., New Haven, CT, United States; ^4^ Department of Drug Discovery Medicine, Pathology Division, Graduate School of Medicine, Kyoto University, Kyoto, Japan; ^5^ Division of Immunology, Institute for Frontier Life and Medical Sciences, Kyoto University, Kyoto, Japan; ^6^ Shiga University of Medical Science, Otsu, Japan

**Keywords:** antibody-mediated rejection (AMR), liver transplantation, complement 5, eculizumab, donor-specific antibody (DSA)

## Abstract

Antibody-mediated rejection (AMR) remains a refractory rejection after donor-specific antibody (DSA)-positive or blood-type incompatible liver transplantation (LT), even in the era of pre-transplant rituximab desensitization. This is due to the lack of not only effective post-transplant treatments but also robust animal models to develop/validate new interventions. Orthotopic LT from male Dark Agouti (DA) to male Lewis (LEW) rats was used to develop a rat LT-AMR model. LEW were pre-sensitized by a preceding skin transplantation from DA 4–6 weeks before LT (*Group-PS*), while sham procedure was performed in non-sensitized controls (*Group-NS*). Tacrolimus was daily administered until post-transplant day (PTD)-7 or sacrifice to suppress cellular rejections. Using this model, we validated the efficacy of anti-C5 antibody (Anti-C5) for LT-AMR. *Group-PS+Anti-C5* received Anti-C5 intravenously on PTD-0 and -3. *Group-PS* showed increased anti-donor (DA) antibody-titers (*P <*0.001) and more C4d deposition in transplanted livers than in *Group-NS* (*P <*0.001). Alanine aminotransferase (ALT), alkaline phosphatase (ALP), total bile acid (TBA), and total bilirubin (T-Bil) were all significantly higher in *Group-PS* than in *Group-NS* (all *P <*0.01). Thrombocytopenia (*P <*0.01), coagulopathies (PT-INR, *P* =0.04), and histopathological deterioration (C4d+h-score, *P <*0.001) were also confirmed in *Group-PS*. Anti-C5 administration significantly lowered anti-DA IgG (*P <*0.05), resulting in decreased ALP, TBA, and T-Bil on PTD-7 than in Group-PS (all *P <*0.01). Histopathological improvement was also confirmed on PTD-1, -3, and -7 (all *P <*0.001). Of the 9,543 genes analyzed by RNA sequencing, 575 genes were upregulated in LT-AMR (*Group-PS vs. Group-NS*). Of these, 6 were directly associated with the complement cascades. In particular, *Ptx3, Tfpi2, and C1qtnf6* were specific to the classical pathway. Volcano plot analysis identified 22 genes that were downregulated by Anti-C5 treatment (*Group-PS+Anti-C5 vs. Group-PS*). Of these, Anti-C5 significantly down-regulated *Nfkb2, Ripk2, Birc3*, and *Map3k1*, the key genes that were amplified in LT-AMR. Notably, just two doses of Anti-C5 only on PTD-0 and -3 significantly improved biliary injury and liver fibrosis up to PTD-100, leading to better long-term animal survival (*P* =0.02). We newly developed a rat model of LT-AMR that meets all the Banff diagnostic criteria and demonstrated the efficacy of Anti-C5 antibody for LT-AMR.

## 1 Introduction

Antibody-mediated rejection (AMR) is a refractory rejection after donor-specific antibody (DSA)-positive or blood-type incompatible organ transplantation (1). Pre-transplant desensitization with rituximab has dramatically improved the outcome of ABO-incompatible living-donor LT (ABOi-LDLT) by eliminating antibody-producing B lymphocytes, leading to a significant reduction in AMR (2–5). However, once AMR develops, it is still challenging to effectively treat AMR (4, 6), and preformed and *de novo* DSAs aggravate liver transplantation (LT) outcomes (7, 8). Thus, there is an urgent need to develop new interventions for not only pre-transplant prophylactic but also post-transplant therapeutic treatments for the refractory rejection.

The complement system plays an important role in the host defense machinery including innate and adaptive immunity (9). The first complement-targeting agent, eculizumab, is a humanized monoclonal antibody that binds to terminal complement 5 (C5) with high affinity, inhibiting its cleavage to C5a and C5b and preventing the formation of C5b-9, which exerts cytolytic, proinflammatory, and prothrombotic properties (10, 11). Eculizumab has been approved for the treatment of paroxysmal nocturnal hemoglobinuria (12), atypical hemolytic uremic syndrome (13), generalized myasthenia gravis (14), and neuromyelitis optica spectrum disorder (15). Therapies targeting complement pathways have currently been expanding based on various basic research and clinical trials (9, 16, 17).

Current standard treatments for AMR include intensified immunosuppression, plasma exchange, intravenous immunoglobulin, or rituximab administration (6). Since the complement cascades amplify the antigen-antibody reactions, complement-targeted interventions have been attracting attention (18–20), especially in the treatment of AMR after kidney transplantation (21–23). However, the efficacy of complement inhibition on AMR after LT (LT-AMR) remains unclear, due at least in part to the lack of established animal models. We thus aimed to develop a small animal model of LT-AMR, and thereby elucidate the efficacy of anti-C5 antibody against LT-AMR.

## 2 Materials and methods

### 2.1 Animals

Male Dark Agouti (DA) rats (Japan SLC, Inc., Shizuoka, Japan) and male Lewis (LEW) rats (Japan SLC) were used as donors and recipients, respectively. They were housed under specific pathogen-free conditions in a temperature- and humidity-controlled environment with a 12-hour light-dark cycle and allowed free access to tap water and standard chow pellets. All animals received humane care in accordance with the “Guide for the Care and Use of Laboratory Animals” (National Institutes of Health Publication No. 86-23, 2011 revision). All experiments were conducted in accordance with the Animal Research Committee of Kyoto University (MedKyo-19196, -20179, -20549, and -21187).

### 2.2 Experimental design

In Experiment-I, we developed a rat AMR model after Orthotopic LT (OLT), and in Experiment-II, we investigated the therapeutic effect of anti-C5 antibody on AMR using the model.

#### 2.2.1 Experiment-I: development of a rat AMR model

**Figure 1:**
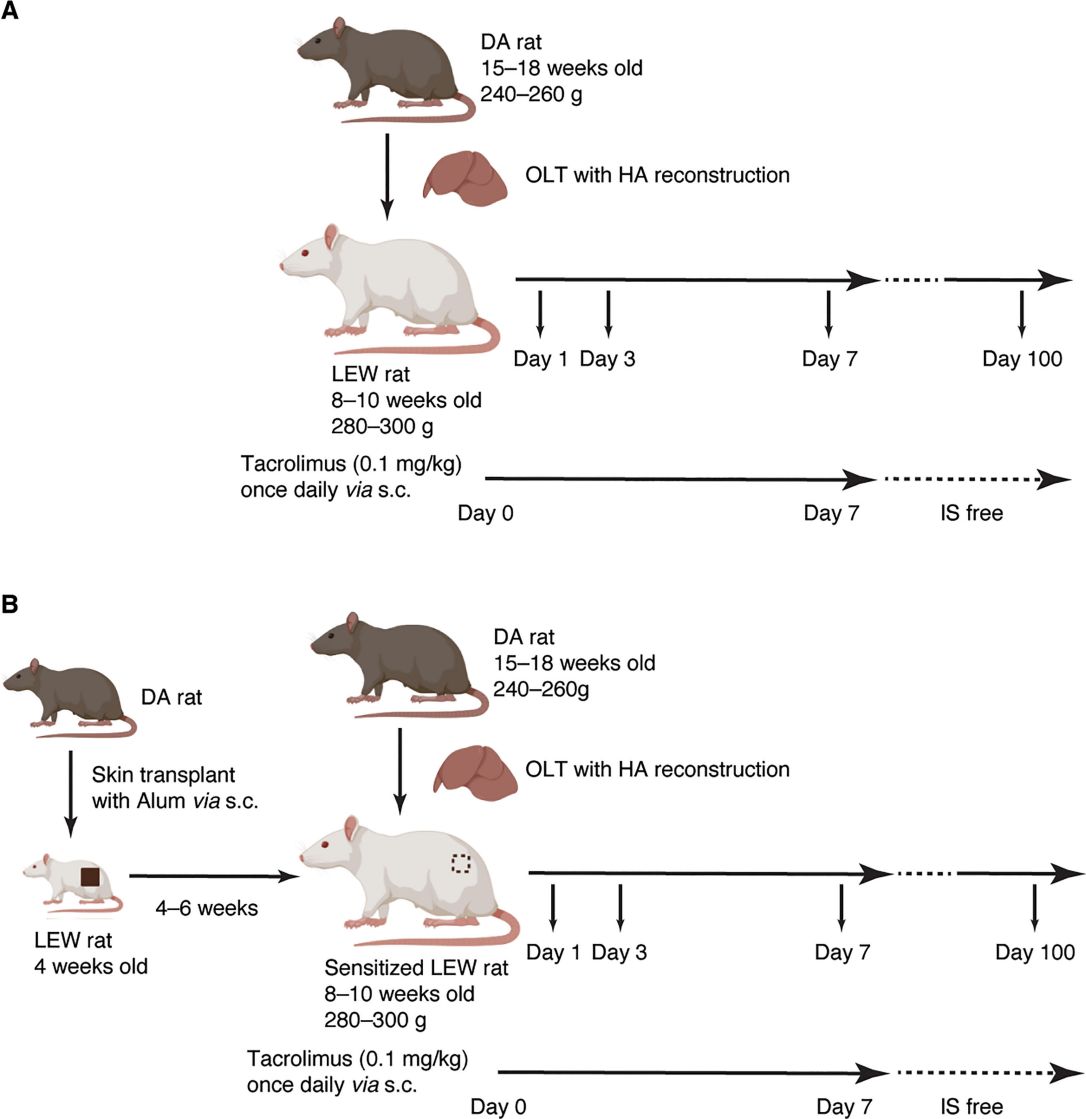
Experimental Design. **(A)** Non-sensitized model (*Group-NS*). OLT with HA reconstruction was performed from DA (15–18 weeks old, 240–260 g) to Lewis rats (8–10 weeks old, 280–300 g). Tacrolimus was daily administered (0.1 mg/kg/day) up to a week or until sacrifice after OLT to regulate T-cell mediated rejection. **(B)** Pre-sensitized model (*Group-PS*). Full-thickness skin grafts (2 x 2 cm^2^) from DA donors were transplanted onto the backs of 4-week-old LEW rats 4–6 weeks prior to OLT. Simultaneously, 50µL of aluminum hydroxide hydrate gel suspension was injected just beneath the skin graft to effectively sensitize B cells. **Figure 1** was created with BioRender.com. DA, Dark Agouti; HA, hepatic artery; IS, immunosuppression; LEW, Lewis; NS, non-sensitized; PS, pre-sensitized; OLT, orthotopic liver transplantation.

OLT with hepatic artery (HA) reconstruction was performed from DA (15–18 weeks old, 240–260 g) to Lewis rats (8–10 weeks old, 280–300 g). In the pre-sensitized group (*Group-PS*), full-thickness skin grafts (2 x 2 cm^2^) from DA were transplanted onto the backs of 4-week-old LEW rats 4–6 weeks prior to OLT (**Figure 1B**), while sham procedure was performed in non-sensitized group (*Group-NS*, **Figure 1A**). Upon skin transplant, 50µL of aluminum hydroxide hydrate gel suspension (ALUM, Cosmo Bio Co., Ltd., Tokyo, Japan) was injected under the skin graft to effectively sensitize B cells (24). After OLT, tacrolimus was daily administered (0.1 mg/kg/day) until post-transplant day (PTD)-7 or sacrifice whichever came first to regulate T-cell mediated rejection (TCMR).

#### 2.2.2 Experiment-II: investigation of the therapeutic effect of anti-C5 antibody on AMR

Monoclonal rat anti-C5 antibody (Anti-C5, TPP-903, 20 mg/kg, Alexion Pharmaceuticals [Cheshire, CT]) was administered intravenously to rats in *Group-PS+Anti-C5* on PTD-0 and -3. Blood and liver tissues were sampled on PTD-1, -3, -7, and -100 in *Group-PS+Anti-C5*. Five replicates at each time point were collected.

### 2.3 Liver donor surgery

The procedures were previously described in detail (25, 26). In brief, donor rats were anesthetized with isoflurane (Isoflurane for Animal, MSD Animal Health K.K., Osaka, Japan) *via* an animal anesthetizer (WP- SAA01, LMS Co., Ltd., Tokyo, Japan). The stereo microscope (SZX10, OLYMPUS Co., Tokyo, Japan) was used for the microsurgery. After midline laparotomy followed by bilateral subcostal incisions, the liver was mobilized from all ligamentous attachments. The right adrenal vein was ligated at its root draining into infra-hepatic inferior vena cava (IHIVC). Common bile duct was cannulated with a 24-gauge ethylene tetrafluoroethylene (ETFE) catheter (TERUMO, Tokyo, Japan) and used as a stent. Pyloric and splenic veins were ligated and dissected, and the portal vein (PV) was isolated. After heparinization (1 IU/g; Mochida Pharmaceutical Co., Ltd., Tokyo, Japan), gastroduodenal artery was ligated. Descending aorta was clamped, and supra-hepatic IVC (SHIVC) was cut in the thoracic space, followed by blood washout with 70 mL of ice-cold University of Wisconsin (UW) solution (Belzer, Bridge to Life Ltd., Northbrook, IL, USA) from the abdominal aorta. The IHIVC and PV were transected, and the HA was removed with an aortic cuff at the root of the celiac artery (CA). The whole liver graft was then retrieved and immediately put in a basin filled with UW solution at 4°C.

### 2.4 Back-table procedure

The back-table procedure was performed on the ice-cold basins. A 7-0 polypropylene suture (PROLENE, Ethicon, Inc., Raritan, NJ, USA) was applied to each bilateral end of the SHIVC. A cuff made from a 14-gauge ETFE catheter (TERUMO) was attached to the PV. Left gastric and splenic arteries were ligated and divided. CA was cut at the root of the abdominal aorta. The graft was stored for 4 hours in UW solution at 4°C.

### 2.5 Recipient surgery

Under general anesthesia with isoflurane, the recipient abdomen was opened through a bilateral subcostal incision. After mobilization of the liver, the right adrenal vein was ligated at its root of IHIVC. The bile duct and proper HA were ligated and dissected at the hepatic hilum. The liver graft was flushed with 5 mL of cold lactated ringer’s solution *via* PV. After injecting 2 mL of lactated ringer’s solution *via* the penile vein, the recipient liver was removed, and the liver graft was placed orthotopically. The SHIVC was anastomosed end-to-end with continuous 7-0 polypropylene sutures. The PV was reconstructed by the cuff technique (27), and the liver was re-perfused within 16 minutes of PV clamp. The IHIVC was then anastomosed in an end-to-end fashion with continuous 8-0 polypropylene sutures. After IHIVC reconstruction, 2 mL of lactated ringer’s solution was injected. The HA was reconstructed by inserting the recipient’s proper HA into the CA orifice of the liver graft, and the CA stump was sutured with 10-0 polyamide monofilament sutures (Kono Seisakusho Co., Ltd., Chiba, Japan). The bile duct was reconstructed by inserting a tube stent.

### 2.6 Sampling

Blood and liver tissues were sampled on PTD-1, -3, -7, and -100 (**Figure 1**). Ethylenediaminetetraacetic acid (EDTA)-contained blood, serum, citric acid-contained plasma, 4% paraformaldehyde (PFA)-fixed liver tissue, frozen liver tissue with Tissue-Tek O.C.T. compound (Sakura Finetek Japan Co., Ltd., Tokyo, Japan.), and frozen liver specimen were obtained. Five replicates at each time point were collected.

### 2.7 Complement hemolytic assay

Terminal complement activity in rat sera was measured by assessing its ability to lyse sheep erythrocytes with the CH50 kit (Denka Company Limited, Tokyo, Japan) according to the manufacture’s protocol. Briefly, each diluted sample was added to diluted sheep erythrocytes in a tube, and then incubated at 37°C for 60 min. After centrifugation, the supernatant was transferred to a microtiter plate, and then absorbance of the supernatant was read at a wavelength of 541 nm using a microplate reader.

### 2.8 Hematology, biochemistry, and coagulation tests

Complete blood count, aspartate aminotransferase (AST), alanine aminotransferase (ALT), alkaline phosphatase (ALP), total bilirubin (T-Bil), direct bilirubin (D-Bil), total bile acid (TBA), and prothrombin time-international normalized ratio (PT-INR) were analyzed (Japan Clinical Laboratories, Kyoto, Japan).

### 2.9 Histological and immunohistochemical analyses

Paraffin-embedded liver tissues (4-µm thickness) were stained with hematoxylin & eosin. Masson trichrome staining was additionally performed on PTD-100 samples. Frozen sections were used for immunostaining with 25-fold diluted anti-rat C4d polyclonal rabbit antibody (HP8034, Hycult Biotech, Uden, Netherlands) overnight at 4 °C without antigen retrieval. The samples were incubated with the Histofine Simple Stain Rat MAX PO (R) (Nichirei Biosciences Inc., Tokyo, Japan) for 30 minutes, followed by washes in phosphate-buffered saline. The stains were visualized with 3,3’-diaminobenzidine tetrahydrochloride solution, counterstained with hematoxylin, and observed with a BZ-9000 microscope (Keyence, Osaka, Japan). The severity of AMR was blindly graded by two independent expert pathologists, according to the AMR diagnostic criteria (28).

### 2.10 Enzyme-linked immunosorbent assay

Serum interleukin (IL)-1b, interferon (IFN)-γ, and tumor necrosis factor (TNF)-α levels were measured using rat Quantikine ELISA Kits (R&D Systems, Minneapolis, MN) according to manufacturer’s instructions.

### 2.11 Measurement of DSAs

Circulating DSAs of IgG and IgM were evaluated in recipient sera by flow cytometry, as described previously (21, 29). Briefly, spleen was removed from DA rats, smashed using Falcon 40µm Cell strainer (Corning Inc., Corning, NY), and hemolyzed using lysing buffer (BD Biosciences, Franklin Lakes, NJ). Fifty µL of aliquots containing 1 x 10^6^ lymphocytes (DA splenocytes) were incubated with 100 µL recipient serum samples diluted (1:80) in phosphate-buffered saline with 1% fetal bovine serum for 60 minutes at room temperature. After 3 washes, cells were labeled with fluorescein isothiocyanate (FITC)-conjugated mouse anti-rat IgG (H+L) secondary antibody (11-4811-85, Thermo Fisher Scientific, Waltham, MA) or FITC-conjugated mouse anti-rat IgM secondary antibody (SA1-25272, Thermo Fisher Scientific). Stained samples were assessed with mean fluorescence intensity on a BD Accuri C6 Flow Cytometer (Becton Dickinson, Mountain View, CA).

### 2.12 RNA sequencing analysis of liver tissues

The RNA-Seq analysis was performed in Azenta Life Sciences (Tokyo, Japan). In brief, 1 µg of total RNA was extracted by the poly(A) mRNA isolation using Oligo(dT) beads for the following library preparation. Libraries with different indexes were multiplexed and loaded on a DNBSEQ-G400 (MGI Tech Co., Ltd., Shenzhen, China) for sequencing using a 2 x150 paired-end configuration according to manufacturer’s instructions. Reference genome sequences and gene model annotation files of relative species were downloaded from the genome website. Data were mapped to the lead sequence obtained with sequence analysis using Hisat2 (v2.0.1), and gene and isoform expression levels were estimated using HTSeq (v0.6.1).

### 2.13 Data analysis of RNA-Seq results

The RNA-Seq data raw counts (11,364 genes in 9 samples) were processed and analyzed with Excel (Microsoft) and the R software package (version 4.2.2). First, genes with expression value less than 10 across all the samples were removed. The remaining 9,543 genes were analyzed using the DESeq2 package (version 1.36.0) (30). Whereby the raw counts were normalized log-transformed and subjected to principal component analysis using the plotPCA function. Differentially expressed genes (DEGs) were calculated with the DESeq2 package, as defined as genes with an adjusted *P*-value <0.05 and a log_2_-fold change >1. Volcano plots were generated using the Enhanced Volcano package (version 1.14.0) (31). The raw counts were normalized using the DESeq2 package, which were then used to construct the clustering heat maps using the heatmap.2 function from the gplots package (version 3.1.3) (32). The clusterProfiler package (version 4.6.2) conducted Gene Set Enrichment Analysis (GSEA) between two groups, using the hallmark gene sets for the *Rattus Norvegicus via* the Molecular Signatures Database. The top 20 hallmark gene sets were graphed and presented as a Dot plot, and the gene sets of interest as a GSEA plot. Finally, for a given hallmark gene set of interest, all the genes listed in the gene set were filtered if they were DEGs, and then graphed as a heatmap. STRING database (version 11.5) was used to find related molecules for the candidate genes. Medium confidence level at 0.400 was used.

### 2.14 Statistical analysis

All data were presented as mean ± standard error of the mean (SEM). Differences among experimental and control groups were analyzed using 2-way analysis of variance (ANOVA), followed by Bonferroni’s *post hoc* tests to assess time-dependent alterations and inter-group differences at each time point. Survivals were estimated by the Kaplan-Meier method, followed by a log-rank test. Student’s P *<*0.05 was considered statistically significant. All statistical analyses were performed with Prism 9 (Graph Pad Software, Inc., San Diego, CA) and R 4.2.2 (https://cran.r-project.org/).

## 3 Results

### 3.1 Experiment-I

#### 3.1.1 Transition of DSAs after skin sensitization

**Figure 2:**
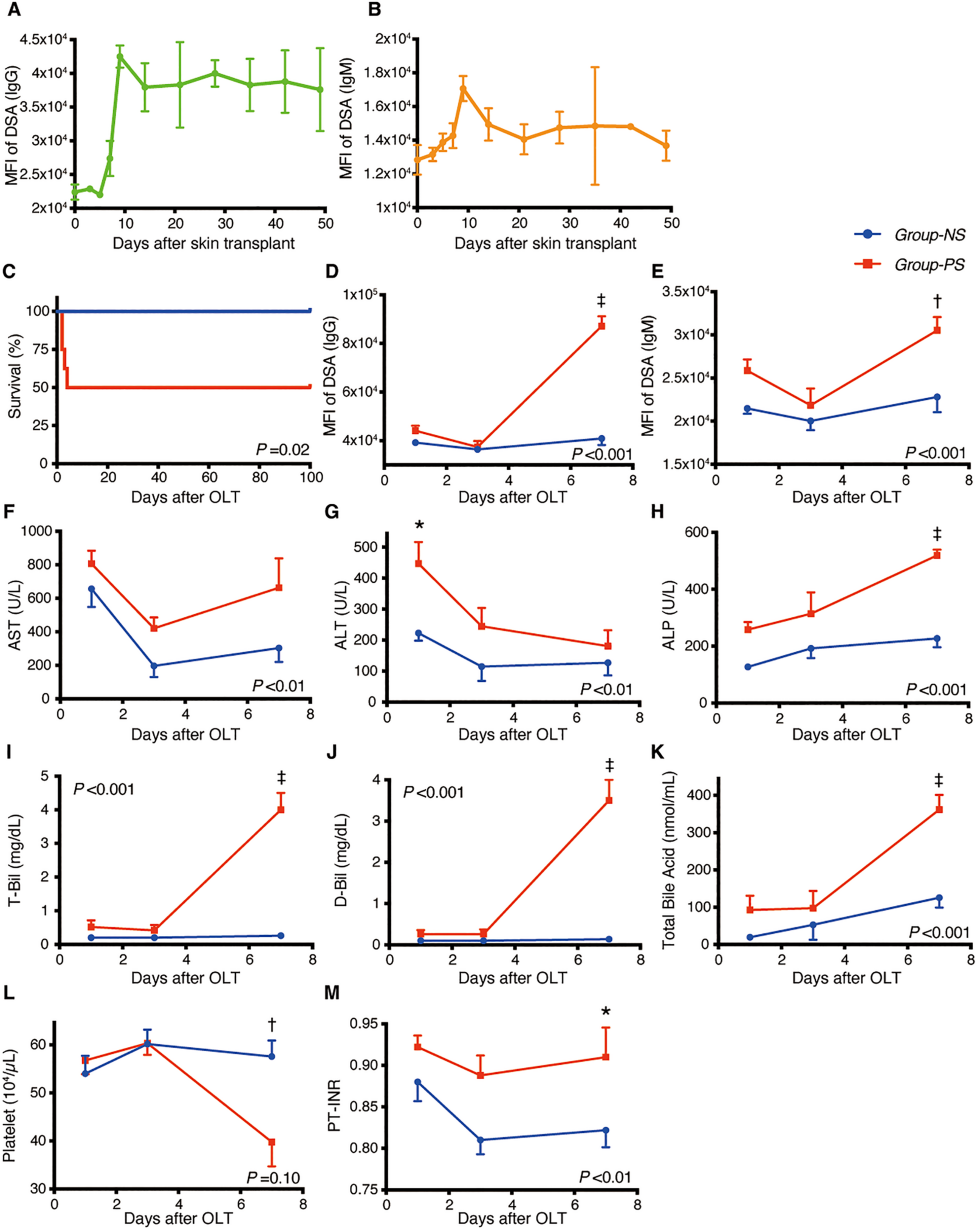
Transition of DSAs, Animal Survival, and Blood Data between *Group-NS* and *Group-PS*. **(A, B)** After pre-sensitization with skin transplants, both IgGand IgM-DSAs peaked on day-9. High MFI-titer of IgG was maintained 7 weeks after skin transplants (*n* =3 at each time point). **(C)** All 8 rats survived up to PTD-100 in *Group-NS*, whereas in *Group-PS*, 4 of the 8 animals died within PTD-4 (*P* = 0.02 by a log-rank test). **(D–M)** All data were given as the mean ± standard error of the mean (SEM, *n* =5 at each time point in both groups). *P*-values by 2-way repeated-measurement ANOVA (intergroup differences) are shown in the lower right or upper left corner of the graphs. *P*-values <0.05 at each time point by Bonferroni’s *post-hoc* tests are summarized as follows: *: *P* <0.05, †: *P* <0.01, ‡: *P* <0.001. ALP, alkaline phosphatase; ALT, alanine aminotransferase; ANOVA, analysis of variance; AST, aspartate aminotransferase; D-Bil, direct bilirubin; DSA, donor-specific anti-human leukocyte antigen antibodies; MFI, mean fluorescence intensity; NS, non-sensitized; OLT, Orthotopic liver transplantation; PS, pre-sensitized; PTD, post-transplant day; PT-INR, prothrombin time and international normalized ratio; T-Bil, total bilirubin.

As shown in **Figures 2A, B**, the preceding skin transplant increased DSA titers of IgG and IgM, both peaking on Day-9 after skin sensitization. High IgG-titers were maintained for 7 weeks.

#### 3.1.2 Animal survival

As shown in **Figure 2C**, all rats survived up to PTD-100 in *Group-NS*, whereas 4 of 8 rats died within PTD-4 in *Group-PS* (*n* = 8 each, *P* = 0.02).

#### 3.1.3 DSAs, biochemistry, hematology, and coagulation tests

*Group-PS* showed significantly higher DSA-titers than *Group-NS* on PTD-7 (MFI of IgG: 87,100 *vs* 40,900, *P <*0.001; MFI of IgM: 30,500 *vs* 22,800, *P <*0.01, **Figures 2D, E**, respectively). Serum transaminase levels were significantly higher in *Group-PS* than in *Group-NS* (ALT: 447 *vs* 223 IU/L, *P* =0.01 on PTD-1, **Figures 2F, G**). Notably, biliary damage markers, i.e., ALP: 519 *vs* 227 IU/L; T-Bil: 4.0 *vs* 0.3 mg/dL; D-Bil: 3.5 *vs* 0.1 mg/dL; and total bile acid: 334 *vs* 133 nmol/mL, were all significantly deteriorated in Group-PS on PTD-7 (all *P <*0.001, **Figures 2H–K**). Moreover, thrombocytopenia (57.6 *vs* 40.0 x 10^4^/µL, *P <*0.01, **Figure 2L**) and coagulation disorders (PT-INR: 0.91 *vs* 0.82, *P* =0.04, **Figure 2M**) were also observed in *Group-PS* on PTD-7 (*n* = 5 at each time point).

#### 3.1.4 Histopathological evaluation

**Figure 3:**
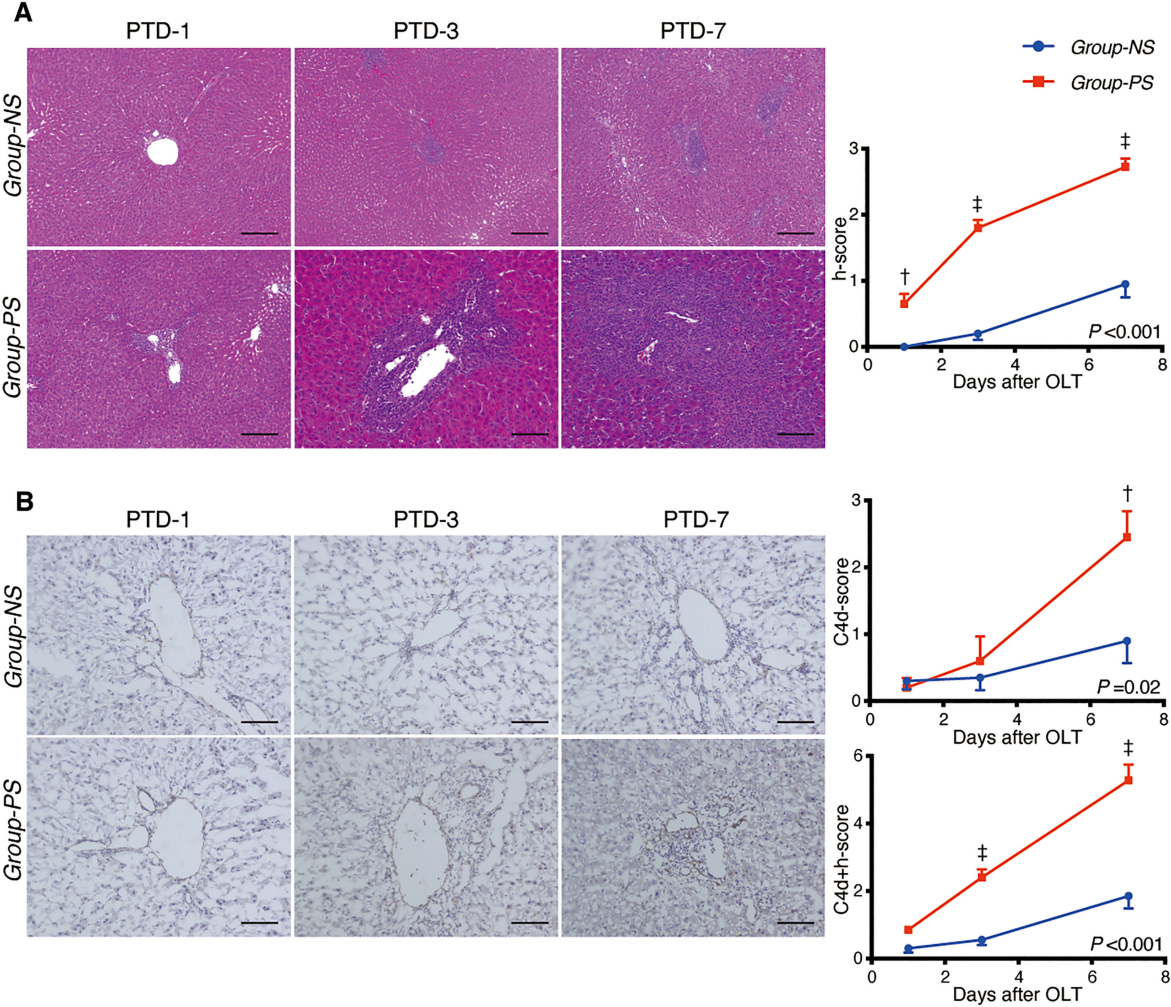
Histopathological Evaluation between *Group-NS vs. Group-PS*. **(A)** Representative tissue sections stained with hematoxylin & eosin from both groups on post-transplant day (PTD)-1, -3, and -7. Histopathology-score (h-score) was significantly higher in *Group-PS* than in *Group-NS* (Intergroup difference by 2-way ANOVA: *P* <0.001; time-point assessment by Bonferroni’s post-tests: ^†^ *P* <0.01 on PTD-1, ^‡^ *P* <0.001 on PTD-3 and -7). Scale bars indicate 100μm. **(B)** Representative C4d immunostaining from both groups on PTD-1, -3, and -7. More C4d deposition in transplanted liver tissues was observed in *Group-PS* than in *Group-NS*, especially in the portal region and sinusoids (C4d-score on PTD-7, ^†^ *P* <0.01). C4d+h-score was significantly higher in Group-PS than in *Group-NS* (^‡^ *P* <0.001 on PTD-3 and ^‡^ *P* <0.001 on PTD-7). Scale bars indicate 100μm. NS, non-sensitized; OLT, Orthotopic liver transplantation; PS, pre-sensitized; PTD, post-transplant day.

As shown in **Figure 3A**, liver tissues were significantly deteriorated in *Group-PS* compared to those in *Group-NS* (histopathology-score [h-score]: 0.7 *vs* 0.0, *P <*0.01 on PTD-1; 1.8 *vs* 0.2, *P <*0.001 on PTD-3; and 2.7 *vs* 1.0, *P <*0.001 on PTD-7). Also, C4d deposition was significantly more evident in Group-PS than in *Group-NS* (C4d-score: 2.5 *vs* 1.0, *P <*0.01 on PTD-7, **Figure 3B**). Accordingly, C4d+h-score was significantly higher in *Group-PS* than in *Group-NS* (2.4 *vs* 0.6, *P <*0.001 on PTD-3 and 5.3 *vs* 1.9, *P <*0.001 on PTD-7, **Figure 3B**). Taken together, these data demonstrate that *Group-PS* is a model that meets the Banff criteria (28) for AMR diagnosis.

### 3.2 Experiment-II

#### 3.2.1 Verification of the complement inhibitory effect of Anti-C5 antibody

**Figure 4:**
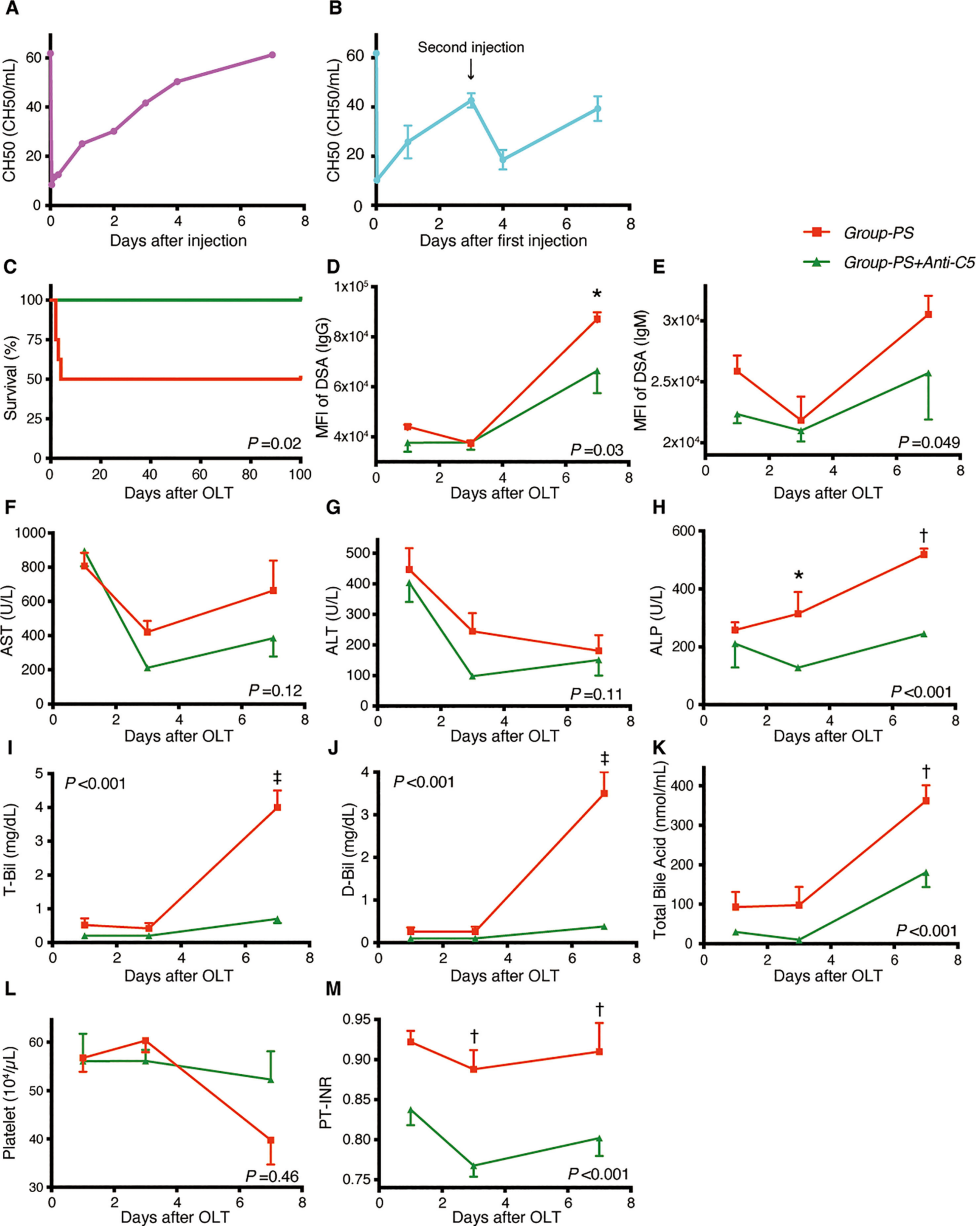
Serum Complement Activities, Animal Survival, and Post-transplant Data: *Group-PS+Anti-C5 vs. Group-PS*. **(A, B)** Rat anti-C5 antibody (TPP-903, 20 mg/kg) was injected intravenously once **(A)** day-0 only) or twice **(B)**, day-0 and -3) into naïve rats, and serum complement activity was determined by CH50 measurement on day-0 (4 and 8 h after injection), -1, -2, -3, -4 and -7. **(C)** All 8 rats survived up to PTD-100 in *Group-PS+Anti-C5*, while 4 of the 8 animals died within PTD-4 in *Group-PS* (*P* = 0.02 by a log-rank test). **(D–M)** All data were given as the mean ± SEM (*n* =5 at each time point in both groups). *P*-values by 2-way repeated-measurement ANOVA (intergroup differences) are shown in the lower right or upper left corner of the graphs. *P*-values <0.05 at each time point by Bonferroni’s *post-hoc* tests are summarized as follows: *: *P* <0.05, ^†^: *P* <0.01, ^‡^: *P* <0.001. ALP, alkaline phosphatase; ALT, alanine aminotransferase; ANOVA, analysis of variance; D-Bil, direct bilirubin; DSA, donor-specific anti-human leukocyte antigen antibodies; MFI, mean fluorescence intensity; NS, non-sensitized; OLT, Orthotopic liver transplantation; PS, pre-sensitized; PTD, post-transplant day; PT-INR, prothrombin time and international normalized ratio; T-Bil, total bilirubin.

First, the complement inhibitory effect of Anti-C5 was verified by measuring the transition of hemolytic activity (CH50). An intravenous injection of 20 mg/kg immediately down-regulated CH50, but the complement activity gradually recovered thereafter and returned the pre-administered value on Day-7 (**Figure 4A**). Therefore, two doses of 20 mg/kg on Day-0 and -3 was then tested (*n* =3 each), demonstrating that complement activity was inhibited and maintained for at least 7 days (**Figure 4B**). Two doses of 20 mg/kg on Day-0 and -3 was thus adopted hereafter.

#### 3.2.2 Animal survival

In *Group-PS*, 4 of 8 rats died within PTD-4 (50% survival); however, Anti-C5 administration significantly improved animal survival to 100% up to PTD-100 (*n* = 8 each, *P* = 0.02, **Figure 4C**).

#### 3.2.3 DSAs, biochemistry, hematology, and coagulation tests

As presented in **Figures 4D, E**, post-transplant Anti-C5 treatment significantly decreased both IgG- and IgM-DSA titers (*P* =0.03 and 0.049, respectively). This amelioration was followed by significant reduction of ALP, T-Bil, D-Bil, total bile acid, and PT-INR, compared to those in *Group-PS* (all *P <*0.001 by 2-way ANOVA). In time-point assessments, *Group-PS+Anti-C5* showed significantly lower IgG-DSA titer than in *Group-PS* (*P* =0.01 on PTD-7, **Figure 4D**). Though statistically not significant, AST and ALT tended to be lowered in *Group-PS+Anti-C5* (**Figures 4F, G**). More importantly, biliary injuries, ALP, T-Bil, D-Bil, and total bile acid, were all significantly ameliorated in *Group-PS+Anti-C5* than in *Group-PS* (all *P <*0.001). Of these, the therapeutic effect on PTD-7 was particularly prominent (ALP: 245 *vs* 519 IU/L, *P <*0.01; T-Bil: 0.7 *vs* 4.0 mg/dL, *P <*0.001; D-Bil: 0.4 *vs* 3.5 mg/dL, *P <*0.001; and total bile acid: 181 *vs* 363 nmol/mL, *P <*0.01, **Figures 4H–K**, respectively). Coagulation disorders (PT-INR) was also significantly improved (**Figures 4L, M**).

#### 3.2.4 Histopathological evaluation

**Figure 5:**
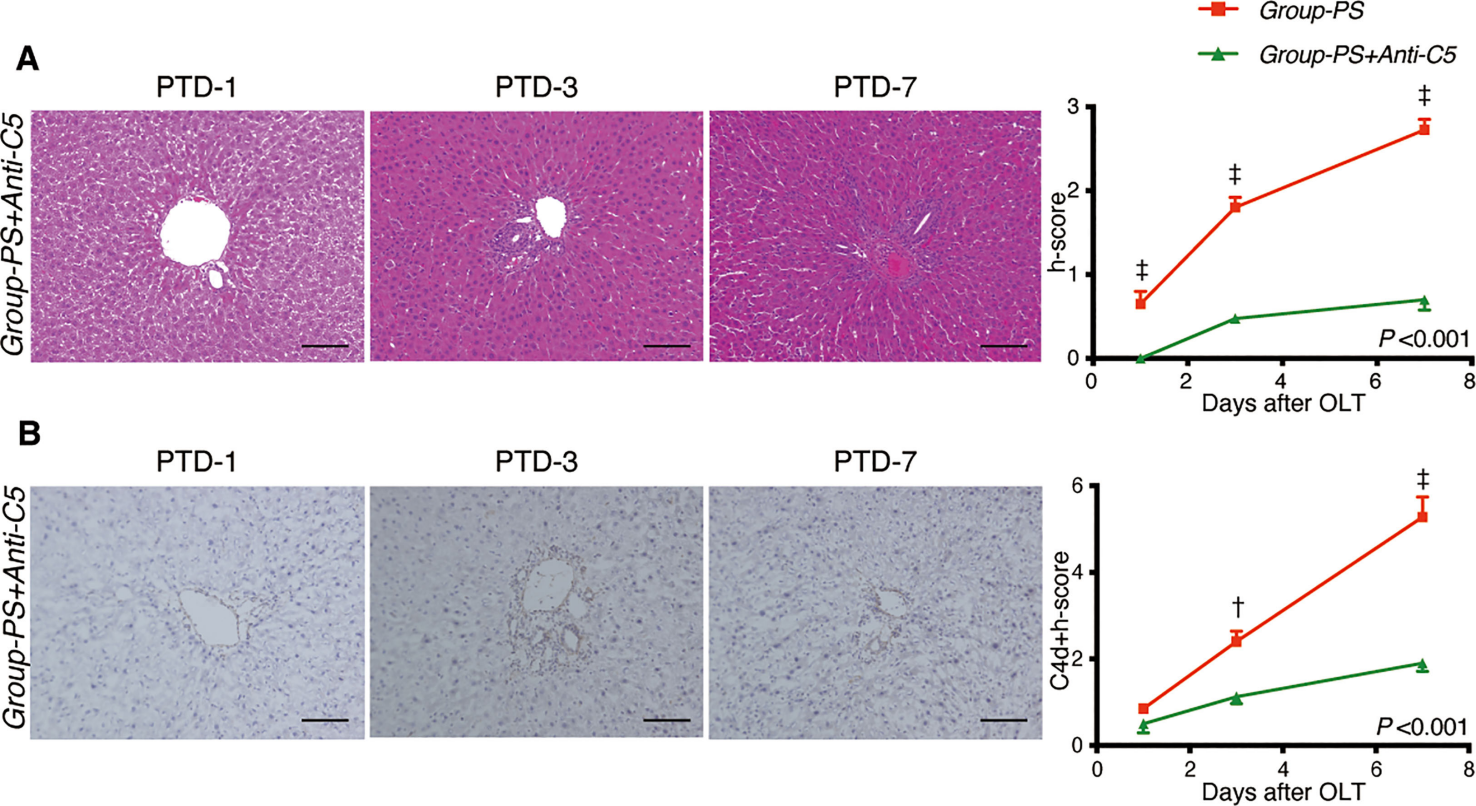
Histopathological Evaluation: *Group-PS vs. Group-PS+Anti-C5*. **(A)** Representative tissue sections stained with hematoxylin & eosin in *Group-PS +Anti-C5* on PTD-1, -3, and -7. Anti-C5 antibody significantly reduced h-score compared to *Group-PS* (**Figure 3**), i.e., pre-sensitized but untreated controls, (Intergroup difference: *P* <0.001; time-point assessment by post-tests: ^†^ *P* <0.01 on PTD-1, ^‡^ *P* <0.001 on PTD-3 and -7). Scale bars indicate 100μm. **(B)** Representative C4d immunostaining in *Group-PS+Anti-C5* on PTD-1, -3, and -7. As seen, C4d deposition was significantly attenuated by post-transplant anti-C5 administration compared to *Group-PS* (**Figure 3**). C4d+h-score was thus significantly lowered in *Group-PS+Anti-C5* than that in *Group-PS* (^†^ *P* <0.01 on PTD-3 and ^‡^ *P* <0.001 on PTD-7). Scale bars indicate 100μm. NS, non-sensitized; OLT, Orthotopic liver transplantation; PS, pre-sensitized; PTD, post-transplant day.

**Figure 5** illustrates representative tissue sections with H.E. (**Figure 5A**) and C4d staining (**Figure 5B**) in both groups. Anti-C5 administration significantly improved liver tissue damages in *Group-PS* (h-score, *P <*0.001), and significantly attenuated C4d deposition (C4d-score, *P <*0.001). Collectively, C4d+h-score was significantly lowered by Anti-C5 treatment (*P <*0.001).

#### 3.2.5 Complement activity and inflammatory cytokines

**Figure 6:**
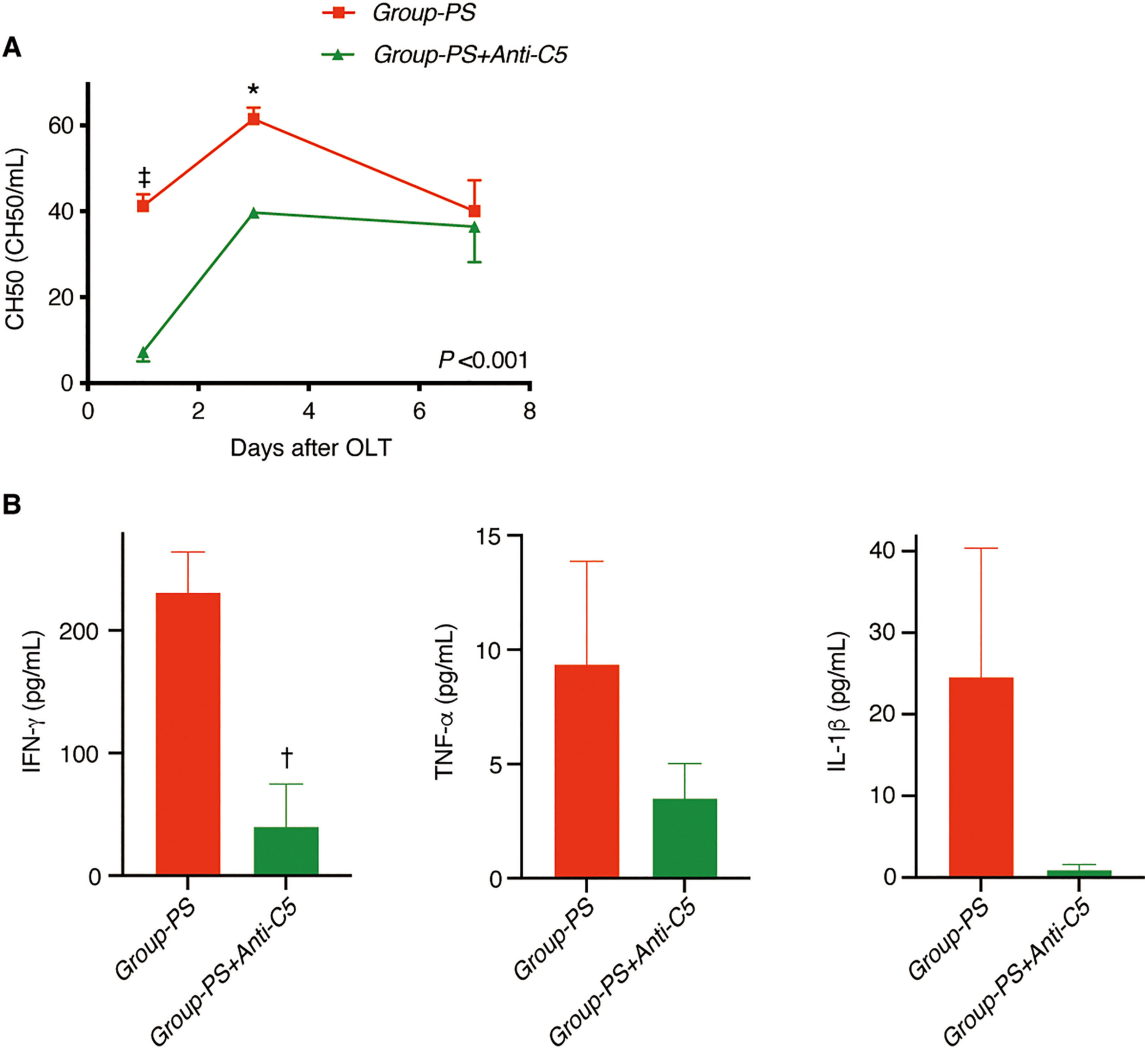
Serum Complement Activities and Inflammatory Cytokines. **(A)** CH50 was significantly lowered in *Group-PS+Anti-C5* than in *Group-PS* (inter-group difference: *P* <0.001 by 2-way ANOVA; time-point assessment by Bonferroni’s post-tests: ^‡^ *P* <0.001 on PTD-1 and *P =0.02 on PTD-3). **(B)** IFN-g was significantly decreased by Anti-C5 administration on PTD-3 (41 vs 231 pg/mL, *P* <0.01). Though statistically not significant, TNF-a and IL-1b were also decreased by Anti-C5 administration on PTD-3. Student’s t-test was conducted to compare two groups. ANOVA, analysis of variance; IFN, interferon; IL, interleukin; OLT, Orthotopic liver transplantation; PS, pre-sensitized; PTD, post-transplant day; TNF, tumor necrosis factor.

CH50 was significantly lower in *Group-PS+Anti-C5* than in *Group-PS* (*P <*0.001 by 2-way ANOVA, 7.3 *vs* 41.2 CH50/mL on PTD-1, *P <*0.001 and 39.7 *vs* 61.5 CH50/mL on PTD-3, *P* =0.02, **Figure 6A**).

Consistently, serum IFN-γ was significantly decreased by Anti-C5 administration on PTD-3 (41 *vs* 231 pg/mL, *P <*0.01, **Figure 6B**). Though statistically not significant, TNF-α and IL-1β also showed similar trend (**Figure 6B**).

#### 3.2.6 Upregulated DEGs in Group-PS compared to Group-NS

Of the 9,543 genes analyzed, we identified 575 DEGs upregulated in LT-AMR on PTD-3 (*Group-PS vs. Group-NS*), 12 of which were involved in the complement functions according to the Rat Genome Database (33). As listed in **Suppl. Table 1**, 6 genes were directly associated with the complement cascades. In particular, *Ptx3*, *Tfpi2*, and *C1qtnf6*, were specific to the classical pathway.

#### 3.2.7 Gene set enrichment analysis

**Figure 7:**
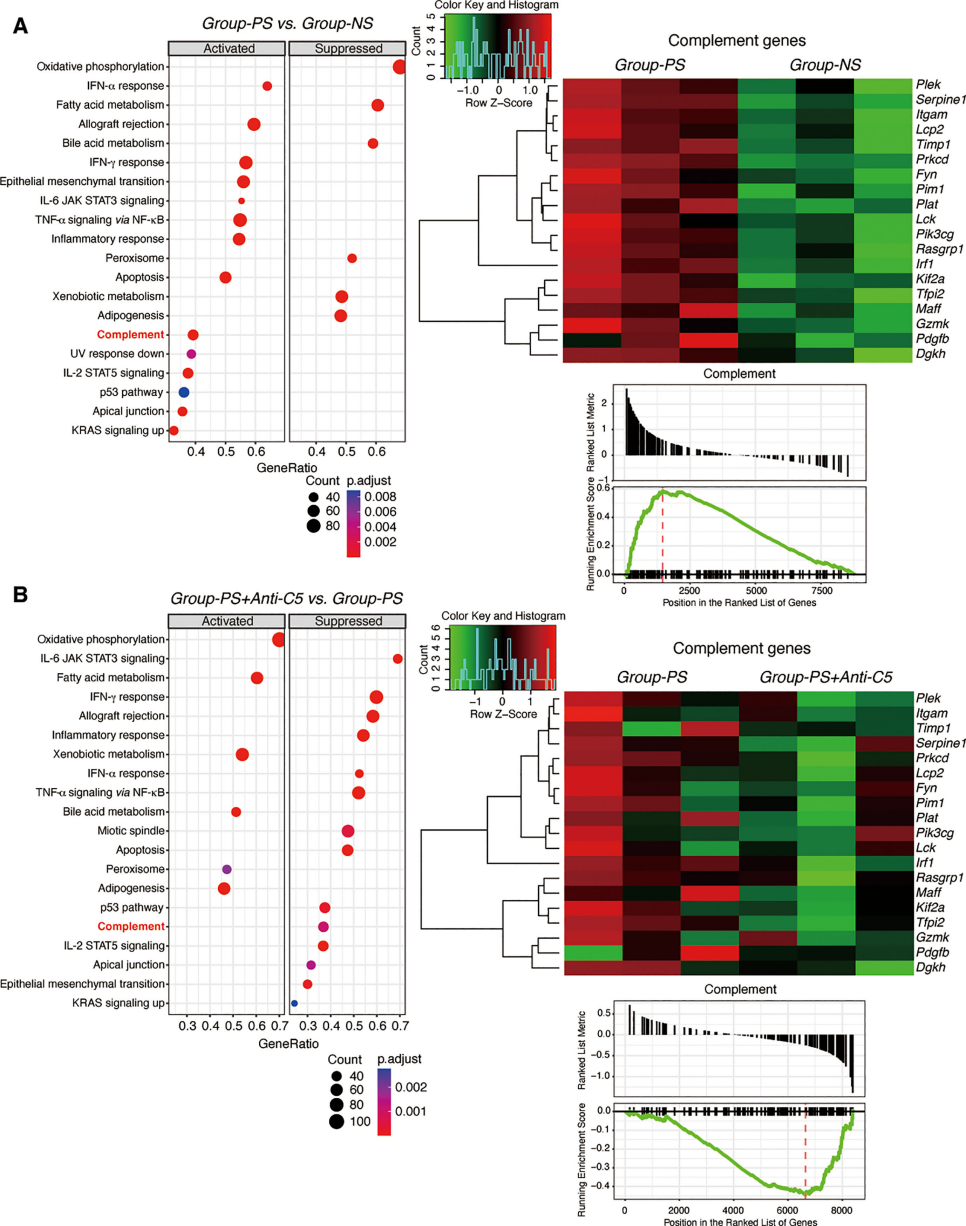
Gene Set Enrichment Analysis. **(A)** RNA-Seq was performed using 3 replicates of transplanted liver tissues on PTD-3 in *Group-NS* and *Group-PS*. From the top 20 significant gene sets enriched; 14 sets including Complement gene set were activated in *Group-PS* and 6 suppressed. The DEGs from the Complement gene set showed that all DEGs were upregulated in *Group-PS*. **(B)** RNA-Seq was performed using 3 replicates of transplanted liver tissues on PTD-3 in *Group-PS* and *Group-PS+Anti-C5*. Anti-C5 treatment suppressed Complement gene set. When the activated complement-associated genes in *Group-PS* were compared to the *Groups-PS+Anti-C5*, a trend of downregulation was observed. IFN, interferon; IL, interleukin; JAK, Janus kinase; KRAS, Kirsten rat sarcoma virus; NES, normalized enrichment score; NF, nuclear factor; NS, non-sensitized; PS, pre-sensitized; STAT, signal transducer and activator of transcription; UV, ultraviolet.

Liver specimens on PTD-3 were used for RNA-Seq to evaluate complement-related genes. MSigDB GSEA was carried out between *Groups-PS* and *Group-NS*. Of the top 20 significant (adjusted *P*-value <0.05) gene sets enriched, 14 were activated in *Group-PS* (of interest, Complement, IFN-α response, Allograft rejection, IFN-γ response, and TNF-α signaling *via* NF-κB) and 6 were suppressed (top gene set, Oxidative phosphorylation, **Figure 7A**, **Supplemental Table 2**). On the other hand, the MSigDB GSEA results comparing *Groups-PS+Anti-C5* and *Group-PS* revealed that Anti-C5 treatment suppressed Complement, IFN-α response, Allograft rejection, IFN-γ response, and TNF-α signaling *via* NF-κB, and activated Oxidative phosphorylation (**Figure 7B**, **Supplemental Table 3**). A deeper analysis was carried out for the MSigDB Complement gene set, and the DEGs therein were filtered and graphed as a heatmap. This displayed that all DEGs were upregulated in Group-PS (**Figure 7A**). When these activated complement-associated genes in *Group-PS* were compared to those in *Groups-PS+Anti-C5*, a trend of downregulation was observed in *Groups-PS+Anti-C5* (**Figure 7B**).

#### 3.2.8 Downregulated DEGs in *Group-PS+Anti-C5* compared to Group-PS

**Figure 8:**
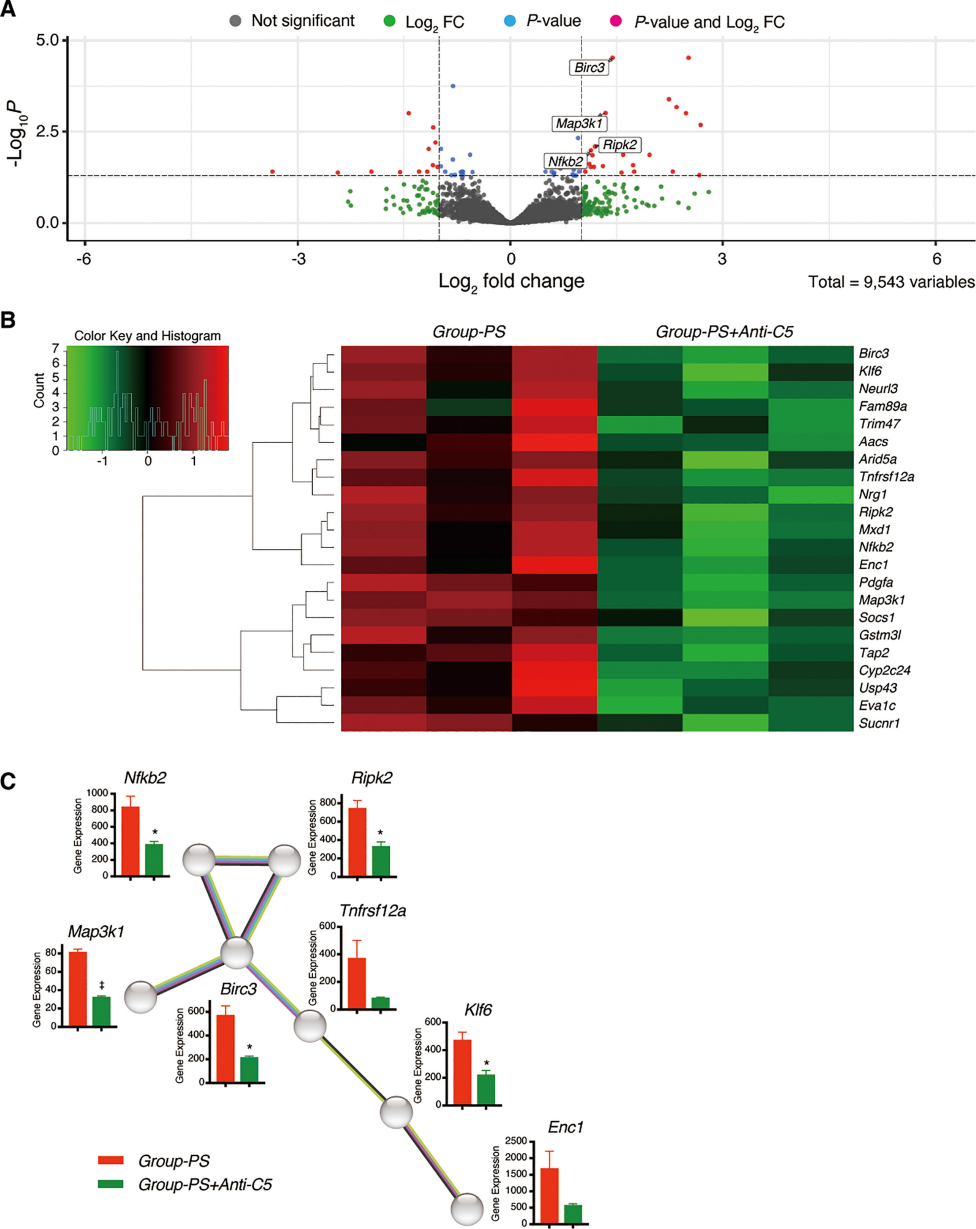
Downregulated differentially expressed genes in *Group-PS+Anti-C5* compared to *Group-PS*. **(A)** RNA-Seq analyses were performed using 3 replicates of transplanted liver tissues on PTD-3 in *Group-PS* and *Group-PS+Anti-C5*. Of the 9,543 genes analyzed, volcano plot analysis identified 22 downregulated genes in *Group-PS+Anti-C5* compared to those in *Group-PS*. **(B)** Heatmap analysis demonstrated *Group-PS+Anti-C5* was clearly distinguished from *Group-PS* by 22 genes identified. **(C)** The STRING database analysis showed that seven genes, *Nfkb2*, *Ripk2*, *Birc3*, *Map3k1*, *Tnfrsf12a*, *Klf6*, and *Enc1* were highly associated with at least one of them. Of these, *Group-PS+Anti-C5* showed significantly lower gene expression by DESeq2 normalized counts in *Nfkb2*, *Ripk2*, *Birc3*, *Map3k1*, and *Klf6* than *Group-PS*. FC, fold change; PS, pre-sensitized; RNA-Seq, RNA sequencing.

Of the 9,543 genes analyzed, volcano plot analysis identified 22 downregulated genes in *Group-PS+Anti-C5* compared to *Group-PS* on PTD-3 (**Figure 8A**, **Table 1**). Heat map analysis revealed that these genes clearly distinguish *Group-PS+Anti-C5* from *Group-PS* (**Figure 8B**). The STRING database analysis (https://string-db.org) showed that seven genes, *Nfkb2*, *Ripk2*, *Birc3*, *Map3k1*, *Tnfrsf12a*, *Klf6*, and *Enc1* were highly associated with at least one of them (**Figure 8C**). Of these, gene expressions by DESeq2 normalized counts in *Nfkb2*, *Ripk2*, *Birc3*, *Map3k1*, and *Klf6* were significantly down-regulated in *Group-PS+Anti-C5* than those in *Group-PS*, suggesting that regulation of these five and their related genes contributed to the AMR alleviation by Anti-C5.

**Table 1:** Downregulated DEGs in *Group-PS+Anti-C5* compared to *Group-PS*.

Genes	log2 Fold Change	*P*-value	Adjusted *P*-value
*Cyp2c24*	2.686	<0.001	0.002
*Sucnr1*	2.668	<0.001	0.049
*Gstm3l*	2.516	<0.001	<0.001
*Tap2*	2.479	<0.001	<0.001
*Neurl3*	2.347	<0.001	<0.001
*Usp43*	2.293	<0.001	0.04
*Tnfrsf12a*	2.238	<0.001	<0.001
*Socs1*	1.965	<0.001	0.01
*Eva1c*	1.748	<0.001	0.04
*Aacs*	1.733	<0.001	0.03
*Enc1*	1.593	<0.001	0.01
*Fam89a*	1.570	<0.001	0.04
*Birc3*	1.443	<0.001	<0.001
*Map3k1*	1.344	<0.001	<0.001
*Arid5a*	1.307	<0.001	0.03
*Ripk2*	1.195	<0.001	0.008
*Mxd1*	1.181	<0.001	0.03
*Nrg1*	1.159	<0.001	0.01
*Nfkb2*	1.138	<0.001	0.01
*Trim47*	1.136	<0.001	0.03
*Klf6*	1.117	<0.001	0.02
*Pdgfa*	1.058	<0.001	0.04

DEGs were defined as genes with an adjusted *P*-value < 0.05 and a log_2_-fold change > 1.

DEGs, differentially expressed genes; NS, non-sensitized; PS, pre-sensitized.

#### 3.2.9 Long-term evaluation

**Figure 9:**
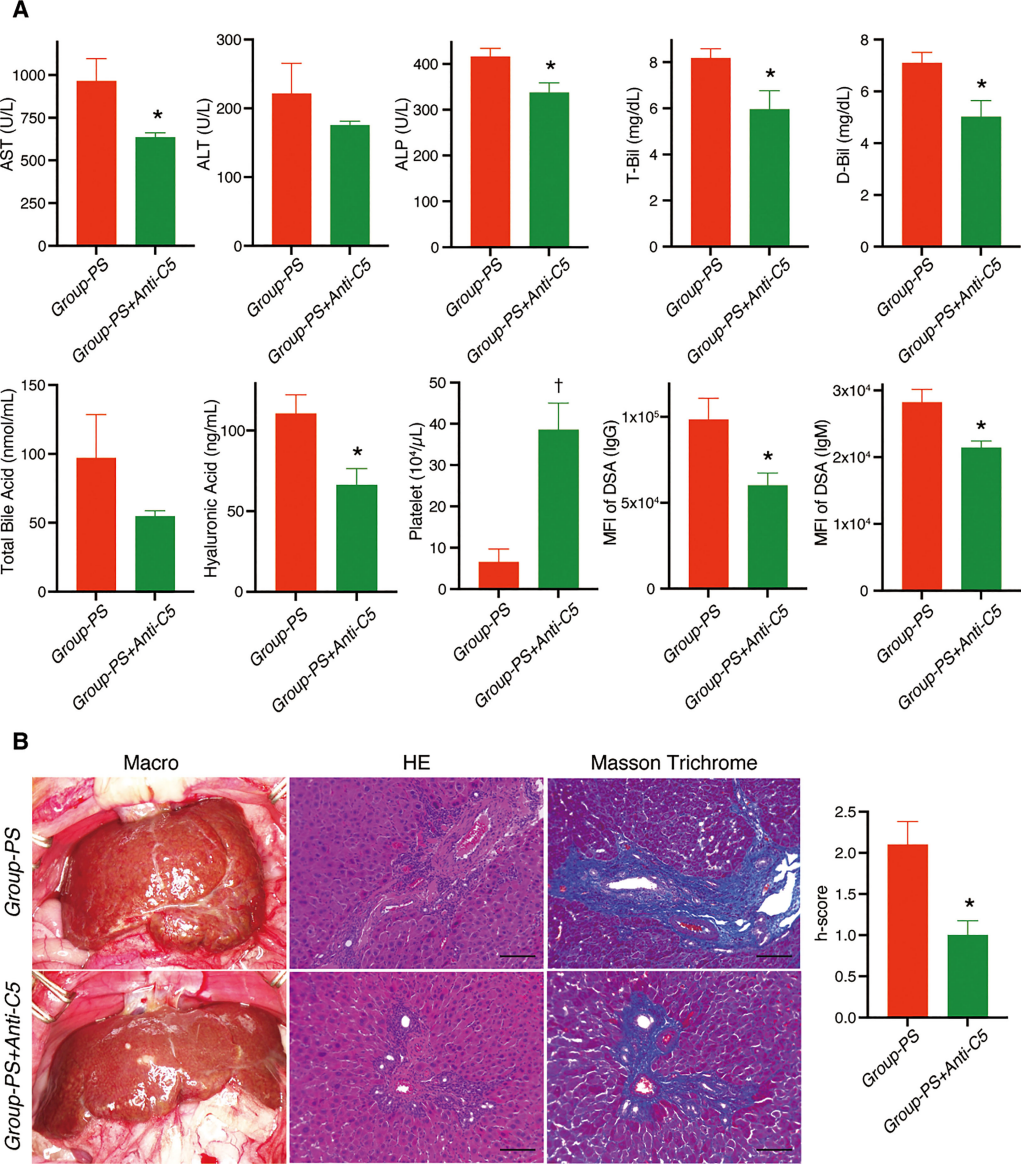
Long-term alterations on PTD-100: *Group-PS+Anti-C5 vs. Group-PS. Student’s t-test was conducted to compare two groups.* **(A)** Just two doses of Anti-C5 administration on PTD-0 and -3 significantly decreased AST (*P* =0.04), ALP (*P* =0.02), T-Bil (*P* =0.04), and D-Bil (*P* =0.02) than those in *Group-PS* even on PTD-100. Hyaluronic acid, a marker for liver fibrosis, was also significantly lowered in *Group-PS+Anti-C5* than in *Group-PS* (*P* =0.02). Of note, peripheral platelet count was 6-times higher in *Group-PS+Anti-C5* than that in *Group-PS* (*P <*0.01). Just 2 doses of Anti-C5 administration on PTD-0 and -3 maintained significantly lower DSA- titers than in *Group-PS* (IgG: *P* =0.03 and IgM: *P* =0.01) at least up to PTD-100. **(B)** Anti-C5 administration significantly attenuated liver fibrosis macro- and microscopically (H.E. and Masson Trichrome staining). Histopathology-score (h-score) was significantly lowered compared with that in *Group-PS* on PTD-100 (*P* =0.01). Scale bars indicate 100µm. *: *P <*0.05, †: *P <*0.01. ALP, alkaline phosphatase; ALT, alanine aminotransferase; ANOVA, analysis of variance; AST, aspartate aminotransferase; D-Bil, direct bilirubin; DSA, donor-specific anti-human leukocyte antigen antibodies; MFI, mean fluorescence intensity; PS, pre-sensitized; PTD, post-transplant day; T-Bil, total bilirubin.

In a long-term evaluation on PTD-100, IgG- and IgM-DSA titers were still significantly lowered in *Group-PS+Anti-C5* than in *Group-PS* (*P* =0.03 and 0.01, respectively). In line, AST (*P* =0.04), ALP (*P* =0.02), T-Bil (*P* =0.04), and D-Bil (*P* =0.02) were also significantly lower in *Group-PS+Anti-C5* than in *Group-PS* (**Figure 9A**). Serum hyaluronic acid, a well-known parameter of liver fibrosis, was significantly lower in *Group-PS+Anti-C5* than in *Group-PS* (*P* =0.02). Severe thrombocytopenia in *Group-PS* (6.5 x 10^4^/µL) was significantly ameliorated by Anti-C5 administration (38.6 x 10^4^/µL, *P <*0.01), and the inter-group difference was more pronounced in the long-term. Consistently, fibrotic/cirrhotic change of transplanted livers was also significantly improved by Anti-C5 treatment both macro- and microscopically (Masson Trichrome staining) with lower h-score (1.0 *vs*. 2.1, *P* =0.01, **Figure 9B**).

## 4 Discussion

To date, several methods of pre-sensitization have been reported in rodent AMR models, including skin (21, 34, 35), splenocyte (29, 36), and blood sensitization (37). All these methods are, however, for AMR models of kidney or heart transplantation, and no robust LT-AMR models have yet to be established thus far. In the present study, we developed a new rodent model of LT-AMR. Since the expected rejection did not occur with splenocyte sensitization (data not shown), we employed skin transplant for pre-sensitization. Unlike sensitization with splenocytes or blood cells, skin transplant is a tissue transplant; therefore, it may strongly sensitize T cells as well as B cells. We thus adopted simultaneous ALUM injection just beneath the skin graft to efficiently sensitize B cells (24). Another unique feature of our LT-AMR model is that the interval from pre-sensitization to LT was set at 4–6 weeks. Although shorter intervals have been reported, e.g., 5 days (36), 1 week (21, 29), and 2 weeks (34), it takes about 2 weeks after immunization for the immune response to peak at germinal centers, the main source of memory B cells and long-lived plasma cells, even in rodents (38). Although the transplanted skin was rejected and spontaneously shed 4 weeks after skin transplants, DSA-IgG remained high in titer after 4 weeks, probably due to the simultaneous ALUM injection. Consequently, our model is, to our knowledge, the first small animal model that meets all the Banff diagnostic criteria for LT-AMR.

Tacrolimus is essential to control TCMR, allowing us to investigate short-term as well as long-term outcomes. Recipient rats not receiving tacrolimus died around 10 days after OLT (39, 40), but all rats in *Group-NS* survived up to 100 days by just one week of daily tacrolimus administration. Since persistent exposure to high DSA titers after LT is associated with increased susceptibility to liver fibrosis in the late post-transplant period (41), *Group-PS*, i.e., preceding skin transplant + simultaneous ALUM injection followed by target organ transplant 4 weeks later, is a useful experimental model to evaluate the impact of DSA on long-term outcomes after OLT. In fact, *Group-PS* represents characteristic biliary damages in LT-AMR, i.e., significant increases of ALP, marked jaundice with D-Bil predominance, and elevated TBA from early to chronic phases, eventually leading to biliary cirrhosis. All these deleterious alterations in intra-hepatic bile ducts were significantly ameliorated by just 2 doses of anti-C5 antibody on PTD-1 and -3, suggesting the importance of complement inhibition in the early phase of AMR development.

It would be of great interest to investigate the detailed mechanisms underlying the significant reduction of DSA titers and its sustained therapeutic effects achieved by early post-transplant anti-C5 treatment. Allograft rejection produces proinflammatory cytokines such as IFN-γ, TNF-α, and IL-1β, which in turn, activate HLA class II molecules expression on vascular endothelial cells (42), drive immunoglobulin class-switch recombination (43), and induce B-cell activation and differentiation to antibody-producing plasma cells (44). Anti-C5 antibody regulates not only inflammatory cytokines but also complement fragment C5a, well-known as a strong anaphylatoxin (16, 45), thereby attenuating AMR and DSA production. Consistently, it has been reported that post-transplant administration of tocilizumab, an anti-IL-6 receptor antibody, alleviates chronic AMR and decreases DSA titers in HLA-sensitized kidney transplant patients, supporting our surmise aforementioned (46). We also demonstrated that just a single dose of Anti-C5 antibody ameliorates not only hepatic ischemia/reperfusion injury (16) but also fulminant hepatitis/acute liver failure in murine models (17), dominantly *via* the C5a-mediated cascade. Since the antigen-antibody reaction and the complement cascades enhance/amplify each other (18–20), early blockade/prophylaxis of such vicious cycle undoubtedly contributes to the sustained reduction of DSA titers. These results suggest the potential of combined use of pre-transplant rituximab and post-transplant Anti-C5 treatment as a promising strategy in high-risk transplants for AMR, e.g., DSA-positive or blood-type incompatible cases.

AMR was effectively treated with anti-C5 antibody in this study. RNA-Seq analysis identified *Nfkb2*, *Ripk2*, *Birc3*, and *Map3k1* as key genes for AMR mitigation by anti-C5 antibody. NF-κB is a central mediator of pro-inflammatory responses and functions in both innate and adaptive immune cells (47). *Ripk2* is essential for NF-κB activation (48). *Map3k1* regulates numerous intracellular signaling pathways involved in inflammation and apoptosis (49). *Birc3* activates the NF-κB and MAPK pathways (50). Collectively, the significant improvement in LT-AMR by anti-C5 antibodies was at least substantially attributable to the attenuation of the inflammatory cycle. In fact, significant down-regulation of IFN-γ, a well-known enhancer of rejection (39), on PTD-3 also supports the above mechanism.

To further investigate the alterations of complement-related genes in LT-AMR and thereby to assess which complement pathway is predominantly involved in LT-AMR, we have analyzed the DEGs in *Group-PS* compared to the *Group-NS*. Of the 575 DEGs upregulated in LT-AMR, 12 of which were involved in the complement cascades. In particular, *Ptx3*, *Tfpi2*, and *C1qtnf6* were specific to the classical pathway. Complement C1 plays a pivotal role in the classical pathway and its antibody-mediated activation is well-known to cause allograft damage *via* activated C1q complex binding to antigen-antibody complexes (1, 18, 51, 52). In fact, C1q binding assays are widely used in clinical practice to identify antibodies with high complement binding capacity (51, 53). Our data, *Ptx3*, *Tfpi2*, and *C1qtnf6* up-regulation in LT-AMR, suggests the significant contribution of the classical pathway in the pathogenesis of LT-AMR.

Of interest, however, most of the 22 DEGs identified in the comparison of *Group-PS+Anti-C5* and *Group-PS* were not specific to the complement pathways and associated with inflammation (**Figure 8**). Since hepatocytes are responsible for the biosynthesis of 80-90% plasma complement components (54), it may be plausible that complement components produced immediately after LT are relatively less rather than those of the inflammatory storm during the acute phase of AMR. This may be a characteristic phenomenon after LT, and it would be interesting to analyze DEGs in an AMR model after other organ transplants in which liver synthetic function remains normal throughout. Besides, the timing of the measurement is also very important. In this study, we used the liver tissues on PTD-3; however, the results on PTD-1 and -100 may be different from that on PTD-3. Such spatial and temporal analyses after liver and other organ transplant at multiple time-points in both the short- and long-term are of great interest in terms of complement-associated immunology after transplantation, and should be the subject of detailed investigations.

Rituximab dramatically improved the outcomes of ABOi organ transplantations and has become a game changer in ABOi-LDLT (2–5); however, it takes 2-3 weeks after rituximab administration for CD19+CD20- antibody-secreting B cells, e.g., short-lived plasma cells and/or plasmablasts to decrease sufficiently (6). Thus, the same strategy cannot be applied to emergent transplants, such as deceased-donor LTs in general or urgent ABOi-LDLT for acute liver failure, *etc.* Our results suggest that if patients cannot afford to wait 2-3 weeks after receiving rituximab, combined use of prophylactic administration of pre-transplant rituximab and post-transplant eculizumab may be able to suppress AMR.

As for anti-rat C5 antibody, ATM-602 has been used so far with inhibitory activity against mouse, rat, and human C5. Recently, a highly purified form of ATM-602, TPP-903 (ATM-602 rat IgG1) was successfully developed (55). TPP-903, used in this study, exerts strong cross reactivity against not only human but also rodent C5 such as rats and mice. To our knowledge, TPP-903 was the most stable antibody that inhibits rat C5 when we started this study in 2020. More antibodies for rats targeting not only C5 but other complement components would further advance complement research. Further, a long-acting anti-C5 antibody, ravulizumab, has become available for use in humans (56, 57). In the near future, it would be of interest to test long-acting anti-rat C5 antibodies in the current LT-AMR model, especially for both emergent and prophylactic use in a variety of AMR.

The current study has several limitations. First, this is an animal study using a rodent AMR model. The anti-C5 antibody for rats showed efficacy against AMR, but further validation in clinical settings is needed to apply the current results to clinical practice. Second, TCMR may not have been completely controlled by 1-week tacrolimus alone. However, in clinical practice, mixed AMR and TCMR, rather than AMR alone, is more often, implying that this model mimics actual clinical conditions. Third, there are limitations on animal species. Cell surface markers and the immune system are less evident in rats than in mice, which has long been a barrier to a detailed immunological assessment in rats. In contrast to the well-established DA-to-LEW rat model (39, 40, 58), however, a standardized mouse rejection model in LT has not yet been developed. This is why we adopted the DA-to-LEW rat OLT model.

In conclusion, we newly developed a rodent model of LT-AMR that meets all the Banff diagnostic criteria for AMR and thereby demonstrated the efficacy of anti-C5 antibody for the refractory LT-AMR. Complement-targeted therapies may play more important roles in the clinical practice of organ transplantation.

## Data availability statement

The datasets presented in this study can be found in online repositories. The names of the repository/repositories and accession number(s) can be found below: PRJNA946923 (SRA).

## Ethics statement

The animal study was reviewed and approved by Animal Research Committee of Kyoto University.

## Author contributions

TTa and KH designed the study. TTa, KH, JK, and HM participated in the performance of the research. TTa, KH, JK, HM, JB, SK, XZ, S-KK, TTs, VK, TW, SU, and EH participated in the analysis and interpretation of data. TTa and KH wrote the manuscript, and EH edited the final version of the draft. KH obtained the research grant. All the authors approved the final version of the article
