# Supplementary material for: Anti-complement 5 antibody ameliorates antibody-mediated rejection after liver transplantation in rats
Source: Front Immunol. 2023 Jun 16;14:1186653. doi: 10.3389/fimmu.2023.1186653 (PMC10313232; doi:10.3389/fimmu.2023.1186653)
Supplement: Supplementary file 1 [file Table_1.docx]

**Supplemental Table 1: Upregulated DEGs directly associated with complement cascade in *Group-PS* compared to *Group-NS***

| Genes | log2 Fold Change | *P*-value | Adjusted *P*-value |
| --- | --- | --- | --- |
| *Fcnb* | 3.099 | <0.001 | <0.001 |
| *Ptx3* | 2.658 | <0.001 | <0.001 |
| *Itgam* | 1.864 | <0.001 | <0.001 |
| *Tfpi2* | 1.699 | <0.001 | <0.001 |
| *Colec12* | 1.593 | <0.001 | <0.001 |
| *C1qtnf6* | 1.284 | <0.001 | 0.001 |

DEGs were defined as genes with an adjusted *P*-value < 0.05 and a log_2_-fold change > 1.

Abbreviations: DEGs, differentially expressed genes; NS, non-sensitized; PS, pre-sensitized.

**Supplemental Table 2: Top 20 significant gene sets enriched in *Group-PS* compared to *Group-NS***

| Gene sets | Enrichment score | NES | Adjusted *P*-value |
| --- | --- | --- | --- |
| Oxidative phosphorylation | -0.677 | -3.129 | <0.001 |
| IFN-α response | 0.614 | 1.786 | <0.001 |
| Fatty acid metabolism | -0.615 | -2.710 | <0.001 |
| Allograft rejection | 0.765 | 2.365 | <0.001 |
| Bile acid metabolism | -0.585 | -2.466 | <0.001 |
| IFN-γ response | 0.683 | 2.126 | <0.001 |
| Epithelial mesenchymal transition | 0.624 | 1.932 | <0.001 |
| IL-6 JAK STAT3 signaling | 0.704 | 2.022 | <0.001 |
| TNF-α signaling via NF-κB | 0.680 | 2.132 | <0.001 |
| Inflammatory response | 0.714 | 2.208 | <0.001 |
| Peroxisome | -0.507 | -2.161 | <0.001 |
| Apoptosis | 0.617 | 1.901 | <0.001 |
| Xenobiotic metabolism | -0.497 | -2.305 | <0.001 |
| Adipogenesis | -0.449 | -2.096 | <0.001 |
| Complement | 0.585 | 1.813 | <0.001 |
| UV response down | 0.512 | 1.565 | 0.005 |
| IL-2 STAT5 signaling | 0.636 | 1.973 | <0.001 |
| p53 pathway | 0.478 | 1.485 | 0.007 |
| Apical junction | 0.559 | 1.720 | <0.001 |
| KRAS signaling up | 0.547 | 1.687 | <0.001 |

Abbreviations: IFN, interferon; IL, interleukin; JAK, Janus kinase; KRAS, Kirsten rat sarcoma virus; NES, normalized enrichment score; NF, nuclear factor; NS, non-sensitized; PS, pre-sensitized; STAT, signal transducer and activator of transcription; UV, ultraviolet.

**Supplemental Table 3: Top 20 significant gene sets enriched in *Group-PS+Anti-C5* compared to *Group-NS***

| Gene sets | Enrichment score | NES | Adjusted *P*-value |
| --- | --- | --- | --- |
| Oxidative phosphorylation | 0.648 | 2.935 | <0.001 |
| IL-6 JAK STAT3 signaling | -0.674 | -2.329 | <0.001 |
| Fatty acid metabolism | 0.574 | 2.436 | <0.001 |
| IFN-γ response | -0.720 | -2.849 | <0.001 |
| Allograft rejection | -0.724 | -2.850 | <0.001 |
| Inflammatory response | -0.692 | -2.723 | <0.001 |
| Xenobiotic metabolism | 0.467 | 2.082 | <0.001 |
| IFN-α response | -0.648 | -2.291 | <0.001 |
| TNF-α signaling via NF-κB | -0.632 | -2.514 | <0.001 |
| Bile acid metabolism | 0.541 | 2.186 | <0.001 |
| Mitotic spindle | -0.432 | -1.725 | <0.001 |
| Apoptosis | -0.553 | -2.142 | <0.001 |
| Peroxisome | 0.449 | 1.810 | 0.001 |
| Adipogenesis | 0.481 | 2.170 | <0.001 |
| p53 pathway | -0.458 | -1.816 | <0.001 |
| Complement | -0.446 | -1.751 | <0.001 |
| IL-2 STAT5 signaling | -0.526 | -2.077 | <0.001 |
| Apical junction | -0.453 | -1.753 | <0.001 |
| Epithelial mesenchymal transition | -0.475 | -1.868 | <0.001 |
| KRAS signaling up | -0.438 | -1.696 | 0.004 |

Abbreviations: IFN, interferon; IL, interleukin; JAK, Janus kinase; KRAS, Kirsten rat sarcoma virus; NES, normalized enrichment score; NF, nuclear factor; NS, non-sensitized; PS, pre-sensitized; STAT, signal transducer and activator of transcription; UV, ultraviolet.
